# Near‐infrared spectroscopy of superficial and deep rectus femoris reveals markedly different exercise response to superficial vastus lateralis

**DOI:** 10.14814/phy2.13402

**Published:** 2017-09-15

**Authors:** Shunsaku Koga, Dai Okushima, Thomas J. Barstow, Harry B. Rossiter, Narihiko Kondo, David C. Poole

**Affiliations:** ^1^ Applied Physiology Laboratory Kobe Design University Kobe Japan; ^2^ Departments of Anatomy and Physiology, and Kinesiology Kansas State University Manhattan Kansas; ^3^ Rehabilitation Clinical Trials Center Division of Respiratory & Critical Care Physiology & Medicine Los Angeles Biomedical Research Institute at Harbor‐UCLA Medical Center Torrance California; ^4^ Faculty of Biological Sciences University of Leeds Leeds United Kingdom; ^5^ Applied Physiology Laboratory Kobe University Kobe Japan

**Keywords:** Deep and superficial muscles, heterogeneity, muscle deoxygenation, on‐ and off‐kinetics

## Abstract

To date our knowledge of skeletal muscle deoxygenation as measured by near‐infrared spectroscopy (NIRS) is predicated almost exclusively on sampling of superficial muscle(s), most commonly the *vastus lateralis* (VL‐s). Recently developed high power NIRS facilitates simultaneous sampling of deep (i.e., *rectus femoris*, RF‐d) and superficial muscles of RF (RF‐s) and VL‐s. Because deeper muscle is more oxidative with greater capillarity and sustains higher blood flows than superficial muscle, we used time‐resolved NIRS to test the hypotheses that, following exercise onset, the RF‐d has slower deoxy[Hb+Mb] kinetics with reduced amplitude than superficial muscles. Thirteen participants performed cycle exercise transitions from unloaded to heavy work rates. Within the same muscle (RF‐s vs. RF‐d) deoxy[Hb+Mb] kinetics (mean response time, MRT) and amplitudes were not different. However, compared with the kinetics of VL‐s, deoxy[Hb+Mb] of RF‐s and RF‐d were slower (MRT: RF‐s, 51 ± 23; RF‐d, 55 ± 29; VL‐s, 18 ± 6 s; *P* < 0.05). Moreover, the amplitude of total[Hb+Mb] was greater for VL‐s than both RF‐s and RF‐d (*P* < 0.05). Whereas pulmonary $\dot{V}O_{2}$ kinetics (i.e., on vs. off) were symmetrical in heavy exercise, there was a marked on‐off asymmetry of deoxy[Hb+Mb] for all three sites i.e., MRT‐off > MRT‐on (*P* < 0.05). Collectively these data reveal profoundly different O_2_ transport strategies, with the RF‐s and RF‐d relying proportionately more on elevated *perfusive* and the VL‐s on *diffusive* O_2_ transport. These disparate O_2_ transport strategies and their temporal profiles across muscles have previously been concealed within the “global” pulmonary $\dot{V}O_{2}$ response.

## Introduction

Pulmonary $\dot{V}O_{2}$ kinetics following the onset and offset of heavy intensity cycling exercise are characterized typically as biexponential processes with primary and slow components reflecting the changes in O_2_ consumption in the active leg muscles (Barstow and Mole 1991; Grassi et al. 1996; Rossiter et al. 2002; Koga et al. 2005; Krustrup et al. 2009; Jones et al. 2012; Poole and Jones 2012 **)**. However, these responses represent a vast consortium of muscle and microvascular units each with its own dynamic O_2_ delivery to O_2_ consumption ($\dot{Q}O_{2}$‐to‐$\dot{V}O_{2}$) ratio that dictates local oxygenation/deoxygenation (i.e., oxy/deoxy[Hb + Mb]) and which differs across and within muscles (Koga et al. 2014). The magnitude of this $\dot{Q}O_{2}$‐to‐$\dot{V}O_{2}$ variance among active muscle fibers has implications for exercise intolerance via its influence on regional maintenance of capillary‐to‐myocyte O_2_ flux required for oxidative phosphorylation. Recently developed multichannel time‐resolved near‐infrared spectroscopy (TRS‐NIRS) has revealed pronounced spatial heterogeneity of absolute muscle deoxy[Hb + Mb] (e.g., Chin et al. 2011; Koga et al. 2011, 2014). However, much of our understanding of muscle deoxygenation dynamics following exercise onset and offset in humans is predicated on NIRS measurements in superficial muscle(s) (e.g., *vastus lateralis*, VL, up to ~1.5 cm depth; e.g., DeLorey et al. 2003; Grassi et al., 2003; Ferrari and Quaresima 2012; Grassi and Quaresima 2016).

Not only does superficial muscle consist of proportionally more fast twitch fibers that are less vascularized and oxidative than deeper muscles (such as the deep *rectus femoris*, RF‐d, or *vastus intermedius*, VI) (Johnson et al. 1973), but also superficial fast twitch fibers are activated later in the recruitment hierarchy than deeper oxidative fibers. Investigations in rodent muscles of different fiber type composition reveal slower and less pronounced deoxygenation profiles in deeper slow‐ (Type I) versus superficial fast‐ (Type II) twitch muscles (Behnke et al. 2003; McDonough et al. 2005) indicative of higher $\dot{Q}O_{2}$/$\dot{V}O_{2}$ ratios in the former (Koga et al. 2012). The greater increases in exercise blood flow found using positron emission tomography (PET) in the human VI compared with the VL provide support for extremely different $\dot{Q}O_{2}$/$\dot{V}O_{2}$ ratios in deep versus superficial muscles (Laaksonen et al. 2013; Heinonen et al. 2015). Moreover, because capillary red blood cell velocity increases more with contractions in less oxidative rodent muscles (Dawson et al. 1987) and greater red blood cell velocity is associated with a greater capillary hematocrit (Kindig et al. 2002), there may also be a greater increase in total[Hb + Mb] within superficial than deeper muscle during heavy exercise where both regions are activated.

Preliminary data gathered as proof‐of‐concept for the high‐power TRS‐NIRS technology used herein supports that, during heavy‐intensity cycle exercise, deoxygenation kinetics of the deep RF are slower than in the superficial RF (Koga et al. 2015). This evidence for fundamentally different $\dot{Q}O_{2}$‐to‐$\dot{V}O_{2}$ control in RF‐d raises questions regarding the extent of this heterogeneity between the RF‐d and the routinely measured superficial VL (VL‐s). Absolute measurements of deoxy[Hb + Mb] and total[Hb + Mb] in deep and superficial muscles would also permit unique insights into the relative importance of *perfusive* (via deoxy[Hb + Mb] i.e., related to $\dot{Q}O_{2}$‐to‐$\dot{V}O_{2}$ ratio) versus *diffusive* (via total[Hb + Mb] i.e., related to the area of interface between red cells and capillary endothelium) O_2_ transport processes in each muscle (McDonough et al. 2005).

Our TRS‐NIRS system has increased the depth sensitivity by ~2‐fold such that the dynamic and spatial heterogeneity of quadriceps deoxy[Hb + Mb] and total[Hb + Mb] for both the superficial (VL and RF, up to ~1.5 cm) and deeper (RF‐d, up to ~3 cm) muscles can be resolved (Koga et al. 2015; Okushima et al. 2015). Based upon the collective evidence from animal and human muscles detailed above we tested the hypotheses that, following onset and offset of heavy intensity exercise, the deeper RF‐d versus superficial VL and RF would have: (1) Slower onset and faster offset kinetics of deoxy[Hb + Mb]; (2) A greater reliance on convective O_2_ transport, as reflected in a smaller amplitude of deoxygenation (deoxy[Hb + Mb]); and (3) A lesser reliance on diffusive O_2_ transport, as reflected by a smaller increase in total[Hb + Mb].

With simultaneous sampling of deeper RF‐d and superficial muscles (RF‐s and VL‐s), the present study complements the study of Koga et al. (2015) that was limited within RF muscle group (i.e., RF‐s vs. RF‐d) and thus provides novel knowledge of deoxygenation both across and within the quadriceps muscles following onset and offset of heavy intensity exercise.

## Methods

Thirteen healthy men (age 21 ± 2 years, height 172 ± 6 cm, and weight 62.3 ± 6.4 kg) provided written informed consent to participate, as approved by the Institutional Review Board of Kobe Design University. All procedures complied with the latest revision of the *Declaration of Helsinki* and *Belmont Report*.

### Measurements

#### Time‐resolved near‐infrared spectroscopy

The principles of operation and algorithms utilized by the equipment have been described in detail elsewhere (Oda et al. 1999). Briefly, a high‐frequency light pulser emits light at three wavelengths, and a single‐photon detector measures the light reflected from the material or tissues under the probe at 5 MHz. Measurement of the absorption coefficient, the reduced scattering coefficient, and the mean path length (calculated from the measured mean photon time of flight) allows calculation of absolute deoxy[Hb + Mb] and oxy[Hb + Mb] in *μ*mol/L units, and their sum, total[Hb + Mb]. From these, tissue O_2_ saturation (S_t_O_2_) is calculated using oxy[Hb + Mb]/total[Hb + Mb].

The absolute changes in muscle deoxygenation and oxygenation profiles during heavy‐intensity cycle ergometry were measured in the quadriceps of the dominant leg. A dual channel TRS‐20 (Hamamatsu Photonics K.K., Japan) was used to measure deoxygenation at the distal sites of superficial VL and RF with spacing between irradiation and detection optodes of 3 cm. A single‐channel high‐power TRS‐20SD (Hamamatsu Photonics K.K., Japan; Suzuki et al. 2014) was used to measure deoxygenation in the deep RF with 6 cm optode spacing on the midbelly that included, in some participants, the superficial region of the VI (Koga et al. 2015; Okushima et al. 2015, 2016).

The output frequency of both TRS systems was set to 1 Hz, and averaged post hoc to increase signal to noise, providing 1 measurement every 2 sec. Calibration of both instruments was performed before each test by measuring the response when the input and receiving fibers face each other through a neutral‐density filter in a black tube.

At the end of the exercise test pen marks were made on the skin to indicate the margins of the NIRS optode holders to reposition the probes for subsequent laboratory visits. Adipose tissue thickness (ATT) and muscle thickness was measured using B‐mode ultrasound (Logiq 400, GE‐Yokogawa Medical Systems, Japan) with the participant at rest and seated in an upright position. In order to quantify the influence of ATT on dynamic changes in NIRS signals, we used the ATT correction method of Bowen et al. (2013).

### Exercise tests

For each exercise session*,* participants reported to the laboratory at least 2 h after their last meal. They were asked to avoid caffeine, alcohol, and strenuous exercise for 24 h before the test. The temperature and relative humidity of the laboratory were maintained at 22°C and 50–65% respectively. Participants were familiarized with exercise testing procedures. All exercise tests were performed in the upright position on an electronically braked cycle ergometer (75XL‐III, Combi, Japan).

Initially, an incremental exercise test (20 W/min, constant pedal frequency at 60 rpm) was used to determine peak $\dot{V}O_{2}$, the gas exchange threshold (GET), and assign heavy‐intensity work rates for the constant‐work‐rate exercise tests. A detailed description of the pulmonary gas exchange measurement in our lab has been published elsewhere (Koga et al. 2007).

Constant work‐rates (heavy‐intensity) were calculated for each individual to require a $\dot{V}O_{2}$ equal to 50% of the difference (Δ) between the participant's GET and peak $\dot{V}O_{2}$. Exercise was performed for 6 min and was preceded by 2 min rest and 4 min of unloaded cycling. Participants performed a single transition of heavy‐intensity exercise on a single day. After each exercise transition, participants performed 6 min of unloaded cycling. During separate visits, the participants performed a total of two to three heavy‐intensity exercise transitions.

### Kinetic analysis

Individual responses of pulmonary $\dot{V}O_{2}$ and deoxy[Hb + Mb] during the baseline‐to‐exercise transitions were time‐interpolated to 1 sec intervals, and averaged across each transition for each participant. The response curve of $\dot{V}O_{2}$ was fit by a three‐term exponential function that included amplitudes, time constants, and time delays, using nonlinear least‐squares regression techniques. The computation of best‐fit parameters was chosen by the program (KaleidaGraph) so as to minimize the sum of the squared differences between the fitted function and the observed response.

The first exponential term started with the onset of exercise and the second and third terms began after independent time delays (eq. 1).

(1) $$\begin{array}{cc} H\left(t\right) & =0\;\text{for}\;t<0\\ & 1\;\text{for}\;t\geq 0\\ \dot{V}O_{2}\left(t\right) & =\dot{V}O_{2}\left(\text{BL}\right)+H\cdot \left(t\right)\cdot A_{c}\cdot \left(1-e^{-t/\tau c}\right)\;\text{phase}\;\;1\;\left(\text{cardiodynamic}\;\;\text{component}\right)\\ & +H\cdot \left(t-{\text{TD}}_{p}\right)\cdot A_{p}\cdot \left[1-e^{-\left(t-\mathrm{TDp}\right)/\tau p}\right]\;\text{phase}\;\;2\;\left(\text{primary}\;\;\text{component}\right)\\ & +H\cdot \left(t-{\text{TD}}_{s}\right)\cdot A_{s}\cdot \left[1-e^{-\left(t-\mathrm{TDs}\right)/\tau s}\right]\;\text{phase}\;\;3\;\left(\text{slow}\;\;\text{component,}\;\text{SC}\right) \end{array}$$

where the subscripts c, p, and s refer to cardiodynamic, primary and slow components, respectively; $\dot{V}O_{2}$ (BL) is the unloaded exercise baseline value; *A*_c_, *A*_p_, and *A*_s_ are the asymptotic amplitudes for the exponential terms; *τ*_c_, *τ*_p_, and *τ*_s_ are the time constants; and TD_p_, and TD_s_ are the time delays. Mean response time (MRT) for primary phase of $\dot{V}O_{2}$ was defined as the sum of TD_p_ + *τ*_p_. The phase I $\dot{V}O_{2}$ at the start of phase II (i.e., at TD_P_) was assigned the value for that time (${A_{c}}^{\prime }$). The physiologically relevant amplitude of the primary exponential component during phase II (${A_{p}}^{\prime }$) was defined as the sum of ${A_{c}}^{\prime }+A_{p}$. Because of concerns regarding the validity of using the extrapolated asymptotic value for the SC ($A_{s}$) for comparisons, we used the value of the slow exponential function at the end of exercise [${A_{s}}^{\prime }=\text{value at}\;360\;\text{sec}-\left(\text{BL}+{A_{p}}^{\prime }\right)$.

The recovery pulmonary $\dot{V}O_{2}$ data (off‐transient response) after heavy exercise were analyzed by a three‐exponential component model (e.g., Özyener et al. 2001), which contains a single time delay for the fast and slow exponential terms during the off‐kinetics.

The deoxy[Hb + Mb] data were fit with a monoexponential model (eq. 2):

(2) $$\begin{array}{cc} H\left(t\right) & =0\;\text{for}\;t<0\\ & 1\;\text{for}\;t\geq 0\\ \text{deoxy}{\left[\text{Hb}+\text{Mb}\right]}_{\left(t\right)} & =\text{deoxy}{\left[\text{Hb}+\text{Mb}\right]}_{\mathrm{BL}}+H\cdot \left(t\right)\cdot \\ A\cdot [1-{e^{-\left(t-\mathrm{TD}\right)/}}^{\tau }] \end{array}$$

where deoxy[Hb + Mb]_BL_ is the unloaded exercise baseline value; *A* is the amplitude of the exponential term; *τ* is the time constant and TD represents the initial component time delay. The deoxy(Hb + Mb) data were fit from the time of exercise onset to 180 sec with the mono‐exponential model, since in our previous study (Koga et al. 2015) the deoxy[Hb + Mb] for the RF‐d reached 95% of the primary component in ~180 sec during heavy exercise [i.e., 10 sec time delay plus three time constants (55 sec)].

The TD and *τ* of the deoxy[Hb + Mb] response were summed to provide an indication of the overall dynamics of muscle deoxygenation in the first 180 sec of exercise (mean response time, MRT). Separately, values for deoxy[Hb + Mb], total[Hb + Mb], and StO_2_ were measured from the mean of the last 60 sec at baseline, and the 20 sec immediately before 180 sec and 360 sec during exercise and recovery.

The analysis of deoxy[Hb + Mb] off‐transient kinetics were fit from the time of exercise cessation to 180 sec using the analogous mono‐exponential model as used for the on‐transient response.

### Statistics

Data are presented as means ± SD. Paired‐samples *t*‐tests were used to compare the $\dot{V}O_{2}$ kinetics parameters between the on‐ and off responses to exercise. A repeated‐measures ANOVA [muscle sites and on vs. off‐transitions] was used to explore differences in deoxy[Hb + Mb], total[Hb + Mb], and StO_2_ responses with Tukey's adjusted post‐hoc tests used to locate statistically significant differences. Statistical significance was accepted when *P* < 0.05.

## Results

The ATT of the VL and the RF were 5.1 ± 1.1 and 6.8 ± 1.3 mm, respectively. The average peak $\dot{V}O_{2}$ was 47.9 ± 9.0 mL·kg^−1^·min^−1^.

### $\dot{\text{V}}{\text{O}}_{2}$ on‐ and off‐response kinetics

**Figure 1:**
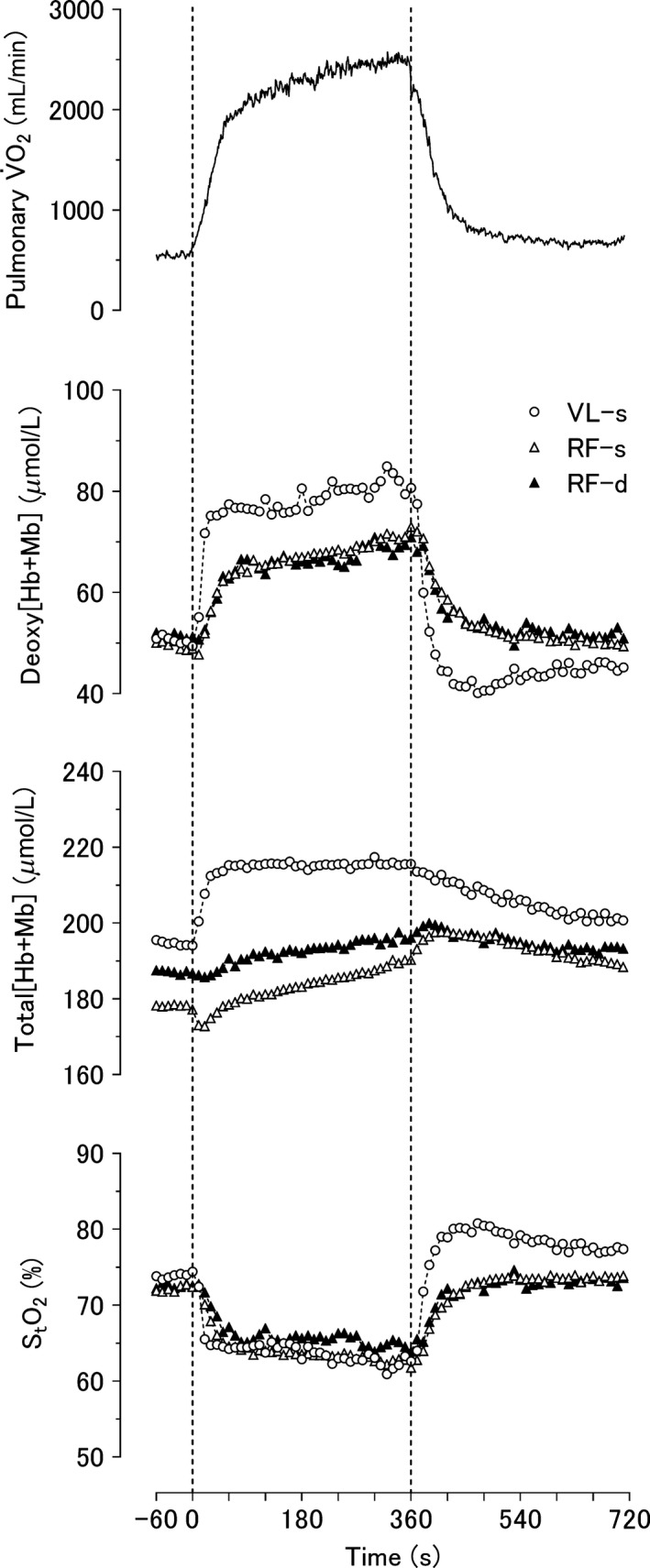
The group mean responses of pulmonary $\dot{V}O_{2}$, deoxy[Hb + Mb], total[Hb + Mb], and StO_2_ following the on‐ and off set of heavy intensity exercise. Exercise onset and offset are at time = 0 and 360 sec, respectively.

The $\dot{V}O_{2}$ on‐ and off‐response kinetics parameters demonstrated close symmetry and conformed to those demonstrated previously for young, healthy individuals (Fig. 1 and Table 1) (e.g., Barstow et al. 1996; Engelen et al. 1996; Carter et al. 2000; Özyener et al. 2001; Poole and Jones 2012).

**Table 1:** Pulmonary $\dot{V}O_{2}$ kinetics following the onset and offset of heavy exercise

	On kinetics	Off kinetics
Baseline, mL·min^−1^	549 ± 32	2484 ± 342*
${A_{c}}^{\prime }$, mL·min^−1^	371 ± 132	373 ± 201
*τ*_p_, sec	28 ± 12	31 ± 10
TD_p_, sec	21 ± 7	20 ± 6
${A_{p}}^{\prime }$, mL·min^−1^	1583 ± 241	1651 ± 256
TD_s_, sec	131 ± 40	20 ± 6*
${A_{s}}^{\prime }$, mL·min^−1^	338 ± 153	211 ± 124
${A_{s}}^{\prime }/\left({A_{p}}^{\prime }+{A_{s}}^{\prime }\right)$	0.17 ± 0.06	0.11 ± 0.06
MRT, sec	49 ± 14	51 ± 8

Values are means ± SD (*n* = 13). $\dot{V}O_{2}$, pulmonary O_2_ uptake; ${A_{c}}^{\prime }$, amplitude of cardiodynamic component; *τ*_p_, time constant of primary component; TD_p_, time delay of primary component; ${A_{p}}^{\prime }$, amplitude of primary component; TD_s_, time delay of slow component; ${A_{s}}^{\prime }$, amplitude of the slow component at end exercise; MRT, mean response time.

*Denotes a significant difference between on and off kinetics (*P *<* *0.05).

### Deoxy[Hb + Mb] on‐response

Within the same muscle (i.e., RF‐d vs. RF‐s) deoxy[Hb + Mb] MRT and amplitude were not different (Fig. 1 and Table 2). However, compared with the VL‐s, deoxy[Hb + Mb] MRT of RF‐s and RF‐d were significantly slower and the RF‐d amplitude was smaller.

**Table 2:** Muscle deoxy[Hb + Mb] kinetics following the onset and offset of heavy exercise

	VL‐s		RF‐s		RF‐d	
	On	Off	On	Off	On	Off
Baseline, *μ*mol/L	50.6 ± 4.9^a^	81.8 ± 16.5^a,^ *	49.5 ± 5.2	70.9 ± 13.2*	51.1 ± 4.8	68.3 ± 6.6*
Amplitude, *μ*mol/L	26.5 ± 14.8^b^	41.3 ± 15.4^a,b,^ *	18.0 ± 9.5	21.7 ± 10.3*	16.4 ± 9.5	17.7 ± 6.4
TD, sec	4 ± 1^a,b^	4 ± 3^a,b^	11 ± 7	13 ± 7	8 ± 7	11 ± 5
*τ*, sec	14 ± 6^a,b^	25 ± 8^a,b,^ *	40 ± 21	64 ± 35*	46 ± 31	59 ± 34*
MRT, sec	18 ± 6^a,b^	29 ± 10^a,b,^ *	51 ± 23	77 ± 37*	55 ± 29	70 ± 32*
Amplitude·*τ* ^−1^, *μ*mol/L·s^−1^	2.09 ± 1.44^a,b^	1.94 ± 1.40^a,b^	0.53 ± 0.37	0.42 ± 0.26	0.42 ± 0.23	0.37 ± 0.21
*R* ^2^	0.95	0.98	0.93	0.93	0.79	0.77

Values are means ± SD (*n* = 13). VL‐s, superficial *vastus lateralis*; RF‐s, superficial *rectus femoris*; RF‐d, deep *rectus femoris* muscle; *R* ^2^, coefficient of determination.

*Denotes the significant difference between on and off kinetics (*P *<* *0.05).

“a” and “b” denote a significant difference between VL‐s and RF‐s and RF‐d, respectively (*P *<* *0.05).

In absolute terms, the initial rate of change in deoxy[Hb + Mb] following the TD (*A*/*τ*) was 4–5 fold greater for VL‐s than RF‐s and RF‐d. The MRT for pulmonary $\dot{V}O_{2}$ primary component for heavy intensity exercise (49 ± 14 sec) was not different from deoxy[Hb + Mb] MRT of RF‐s (51 ± 23 sec) and RF‐d (55 ± 29 sec). Note the presence of a pronounced slow component for the three muscle sites (Fig. 1) as was evident for pulmonary $\dot{V}O_{2}$ (Table 1).

### Total[Hb + Mb] on‐response

The VL‐s total[Hb + Mb] increased to a greater extent, and more rapidly, than the RF‐s and RF‐d (Fig. 1 and Table 3). End‐exercise amplitudes of the total[Hb + Mb] were ~2‐fold greater in VL‐s than RF‐s and RF‐d. However, there were no significant differences in the total[Hb + Mb] amplitude for the RF‐s versus RF‐d.

**Table 3:** The total[Hb+Mb] following the onset and offset of heavy exercise

	VL‐s	RF‐s	RF‐d
On kinetics			
Baseline, *μ*mol/L	194.7 ± 15.8^a^	178.1 ± 13.5	187.3 ± 8.3
Amplitude (0–180 sec), *μ*mol/L	20.7 ± 11.3^a,b^	4.7 ± 9.2	4.9 ± 5.8
Amplitude (0–360 sec), *μ*mol/L	20.8 ± 12.5^a,b^	11.6 ± 9.3	8.9 ± 4.3
Off kinetics			
Baseline, *μ*mol/L	215.8 ± 24.2^a,b,^ *	189.1 ± 16.7*	195.9 ± 10.5*
Amplitude (0–180 sec), *μ*mol/L	9.6 ± 9.3^a,b,^ *	−5.9 ± 10.6*	0.7 ± 6.7
Amplitude (0–360 sec), *μ*mol/L	14.5 ± 8.2^a,b,^ *	−0.1 ± 10.0*	2.7 ± 5.7*

Values are means ± SD (*n *=* *13).

*Denotes the significant difference between on and off kinetics (*P *<* *0.05).

“a” and “b” denote a significant difference between VL‐s and RF‐s and RF‐d, respectively (*P *<* *0.05).

### Off‐responses for deoxy[Hb + Mb] and total[Hb + Mb]

There was a marked on‐off asymmetry in deoxy[Hb + Mb] for the all three muscle sites i.e., MRT‐off > MRT‐on (*P* < 0.05). At exercise cessation, VL‐s deoxy[Hb + Mb] decreased rapidly below baseline (undershoot), whereas RF‐d and RF‐s deoxy[Hb + Mb] recovered more slowly than VL‐s to values close to baseline (Fig. 1).

The total[Hb + Mb] during recovery did not mirror the on‐responses. Specifically, both the RF‐s and RF‐d total[Hb + Mb] actually increased after the end of exercise and remained significantly above baseline throughout the recovery period. Somewhat differently, VL‐s total[Hb + Mb] recovery kinetics were slow relative to the on‐transient, but total[Hb + Mb] recovered ~75% of the way to baseline by 6 min.

### StO_2_ on‐ and off response

There were no significant differences in the StO_2_ on‐amplitude for the three sites. However, the off‐amplitudes for VL‐s were significantly greater than RF‐s and RF‐d. Further, the off‐amplitudes were larger than on‐amplitudes at each site (Fig. 1 and Table 4).

**Table 4:** The S_t_O_2_ following the onset and offset of heavy exercise

	VL‐s	RF‐s	RF‐d
On kinetics			
Baseline, %	73.9 ± 2.9	72.2 ± 2.1	72.7 ± 2.4
Amplitude (0–180 sec), %	9.6 ± 5.1	8.4 ± 3.4	7.1 ± 3.8
Amplitude (0–360 sec), %	11.6 ± 5.6	9.7 ± 3.7	7.5 ± 3.7
Off kinetics			
Baseline, %	62.3 ± 4.1*	62.6 ± 5.0*	65.1 ± 2.9*
Amplitude (0–180 sec), %	17.2 ± 5.0^a,b,^ *	11.1 ± 4.1*	8.1 ± 2.8*
Amplitude (0–360 sec), %	15.2 ± 5.2^b,^ *	11.2 ± 3.7*	8.2 ± 3.2*

Values are means ± SD (*n *=* *13). S_t_O_2_, tissue O_2_ saturation.

*Denotes the significant difference between on and off kinetics (*P *<* *0.05).

“a” and “b” denote a significant difference between VL‐s and RF‐s and RF‐d, respectively (*P *<* *0.05).

## Discussion

Analysis of pulmonary $\dot{V}O_{2}$ kinetics at exercise onset and offset has revealed fundamental characteristics of muscle metabolic control (Whipp and Mahler 1980; Marwood et al. 2011; Rossiter 2011; Poole and Jones 2012) and fitting exponential models to the $\dot{V}O_{2}$ response permits extraction of system parameters (Barstow and Mole 1991; Paterson and Whipp 1991), which reflect the control of O_2_ delivery and utilization predominantly within the active locomotor musculature (Poole et al. 1991; Grassi et al. 1996; Rossiter et al. 2002; Koga et al. 2014; Murias et al. 2014; Benson et al. 2017). However, enshrouded within those parameters is a range of vascular ($\dot{Q}O_{2}$) and metabolic ($\dot{V}O_{2}$) responses that differ across and within muscles depending upon their structure/function and also recruitment profiles (Whipp et al. 1995; Hughson et al. 2001; Kalliokoski et al. 2006; Cannon et al. 2013; Koga et al. 2014; Wüst et al. 2014). Using high‐power TRS‐NIRS, this investigation has, for the first time, resolved profoundly different profiles of muscle deoxygenation and microvascular hemodynamics between the superficial and deep RF, and the VL‐s (the latter being more accessible and most often evaluated muscle region). Specifically, within the same muscle (RF‐s and RF‐d) deoxy[Hb+Mb] kinetics and amplitudes were not different for heavy intensity exercise. However, compared with the VL‐s, the RF‐s and RF‐d have: (1) Slower onset and slower offset kinetics of deoxy[Hb + Mb], suggesting a greater reliance on convective O_2_ transport; and (2) A smaller increase and very slow recovery of total[Hb + Mb], suggesting less reliance on increases in diffusive O_2_ transport. These data expose contrasting O_2_ transport strategies in deeper, and more oxidative, muscles in humans: absolute measurements of deoxy[Hb + Mb] and total[Hb + Mb] provide information of *perfusive* (related to $\dot{Q}O_{2}$‐to‐$\dot{V}O_{2}$ ratio) and *diffusive* (related to the area of interface between red cells and capillary endothelium) O_2_ transport processes in each muscle, respectively, thus supporting that the RF‐s and RF‐d rely proportionately more on elevated *perfusive* O_2_ transport versus *diffusive* O_2_ transport in the VL‐s.

### On‐kinetics of deoxy[Hb + Mb]

Following the onset of the heavy intensity exercise slower deoxy[Hb + Mb] primary component kinetics (*τ* and MRT) for the RF‐d versus superficial VL indicates a higher $\dot{Q}O_{2}$‐to‐$\dot{V}O_{2}$ ratio consistent with maintenance of higher microvascular partial pressure of O_2_ (P*mv*O_2_) (Fig. 1 and Table 2; Behnke et al. 2002; McDonough et al. 2005). These findings are in substantial agreement with preliminary data from our laboratory for superficial versus deep RF (i.e., RF‐d, Koga et al. 2015).

One explanation for these findings is that deeper muscle regions contain proportionally more slow‐twitch (type I) fibers (Johnson et al. 1973). Certainly within highly differentiated slow‐ versus fast‐twitch animal muscles the P*mv*O_2_ on‐contractions response (i.e., slower kinetics with less of a drop in P*mv*O_2_) is consistent with the high vasodilatory control sensitivity in these muscles (Behnke et al. 2003; McDonough et al. 2005; Laughlin et al. 2012). Because human muscles usually constitute a fiber type mosaic rather than the single‐fiber type predominance found in select animal muscles, human muscle‐specific differences would not be expected to be as pronounced as shown in animals (Saltin and Gollnick 1983; Delp and Duan 1996).

However, while the VL‐s and RF‐d deoxy[Hb + Mb] kinetics (*τ* and MRT) differed widely, the small numerical differences between the superficial and deep RF muscle did not reach significance, which was inconsistent with our preliminary study (Koga et al. 2015). High‐power deep tissue TRS‐NIRS aggregates both deep and superficial muscle regions. Therefore, the overall response will be intermediate between the two fiber populations and weighted to surface tissues (Koga et al. 2015). The lack of significant difference between RF‐s and RF‐d deoxy[Hb + Mb] kinetics may reflect that the fiber type and/or activation profiles of the RF muscle were more similar between deep and superficial regions in the present study than in our previous participants, and suggest that the significant differences between the VL‐s and RF‐d deoxy[Hb + Mb] kinetics are not due to the depth of the muscles, rather to the group of muscle being examined (VL vs. RF).

Despite these considerations, the present investigation demonstrates that muscle contractions induce a markedly slower deoxygenation profile in deep tissues than previously thought based solely on superficial NIRS measurements. This finding coheres with PET measurements during moderate exercise of greater blood flow in the VI muscle, which is anatomically deep compared to the other three muscles of *m. quadriceps femoris* (Heinonen et al. 2015). That the VI is located nearest the femur and contributes primarily to resist gravity and assist in body postural control perhaps provides some explanation for the high blood flow especially during low‐to‐moderate intensity exercise. Greater $\dot{Q}O_{2}$ at any given $\dot{V}O_{2}$ would result in lower fractional O_2_ extraction and consequently a greater microvascular P*mv*O_2_ thereby facilitating blood‐myocyte O_2_ flux and potentially optimizing $\dot{V}O_{2}$ kinetics and metabolic control.

### Off‐kinetics of deoxy[Hb + Mb]

Whereas both muscle $\dot{V}O_{2}$ and $\dot{Q}O_{2}$ change synchronously from the onset of contractions in the rat spinotrapezius (Behnke et al. 2002; Kindig et al. 2002) this is not the case during recovery (McDonough et al. 2001, 2004) where $\dot{V}O_{2}$ falls extremely rapidly compared with $\dot{Q}O_{2}$ (Behnke et al. 2009). This asymmetry has been attributed to dissociation between the intracellular biochemical milieu controlling $\dot{V}O_{2}$ and the vasodilatory stimuli regulating $\dot{Q}O_{2}$. Such behavior is also seen in humans where, after cessation of exercise, the kinetics of superficial VL muscle capillary blood flow (Ferreira et al. 2005) and cardiac output (Paterson and Whipp 1991; Yoshida and Whipp 1994) are both slower compared with exercise onset. Although faster on and slower off muscle $\dot{Q}O_{2}$ kinetics, relative to $\dot{V}O_{2}$, would help maintain an elevated P*mv*O_2_ under each circumstance (expressed herein as lower deoxy[Hb + Mb]), and facilitate diffusional blood‐myocyte O_2_ flux, recovery deoxy[Hb + Mb] kinetics were consistently slower than seen following exercise onset across all muscles and regions studied (Fig. 1 and Table 2).

Our hypothesis that the deep muscle would exhibit faster deoxy[Hb + Mb] kinetics than superficial muscles during recovery was predicated upon our previous observations in healthy rat muscles of contrasting fiber types and also in spinotrapezius muscle of rats in heart failure. Specifically, during recovery after moderate intensity contractions, soleus (slow‐twitch, 84% type I) P*mv*O_2_ recovery was faster compared with that of the peroneal (fast‐twitch, 86% type II) (McDonough et al. 2004). For rats in heart failure, deranged vascular control accelerated the fall in P*mv*O_2_ following the onset of contractions and slowed its recovery upon cessation of contractions (Copp et al. 2010). However, inconsistent with our hypothesis, following heavy intensity exercise the deoxy[Hb + Mb] off‐transient MRT in the RF‐d muscle was greater (i.e. slower recovery) than that of the VL‐s (Fig. 1 and Table 2); with both muscles returning to, or below, the pre‐exercise baselines within the 6 min observation period.

This latter observation that VL‐s deoxy[Hb + Mb] actually fell substantially below the pre‐exercise baseline is interesting and was not seen for the RF‐s and RF‐d (Fig. 1; see the lower on‐amplitudes of VL‐s compared with the off‐amplitudes in Table 2). This is consistent with the findings of Fukuoka et al. (2015) in the superficial quadriceps muscles following heavy exercise and reflects a $\dot{Q}O_{2}$ response that is seemingly dissociated from (i.e., greater than) O_2_ metabolic requirements in VL‐s but not RF‐d. The apparent prolongation of postcontraction vasodilatation (i.e., postexercise hyperemia) in VL‐s is likely driven by residual tissue metabolic perturbations and may serve to remove associated by‐products (e.g., lactate, adenosine, K^+^) that presumably accumulate more in the predominantly glycolytic and lower PO_2_ environment of the VL‐s than the RF‐s and RF‐d during contractions.

A striking observation was that total[Hb + Mb] (indicative of diffusive O_2_ conductance) did not return to baseline within 6 min postexercise in either muscle. While total[Hb + Mb] in the VL‐s recovered approximately 75% of the way to baseline by 6 min, tissue hemoglobin concentration in the RF‐s and RF‐d remained at, or transiently slightly above, the value seen during contractions (Fig. 1). Collectively, these data suggest that $\dot{Q}O_{2}$ does not constrain recovery of baseline deoxy[Hb + Mb] (or $\dot{V}O_{2}$ therefore) for either the RF‐d or the superficial muscles (RF‐s and VL‐s) after heavy exercise (Yoshida and Whipp 1994; McDonough et al. 2001).

### Experimental considerations

Contrasting $\dot{Q}O_{2}$‐to‐$\dot{V}O_{2}$ matching strategies among muscles were revealed by the substantial range of deoxy[Hb + Mb] responses both at the on‐ and off‐transient. For instance, the rate of deoxy[Hb + Mb] change i.e., amplitude/*τ* (*μ*mol/L/sec) in recovery after heavy intensity exercise was greater in VL‐s than both RF‐s and RF‐d (1.94 *μ*mol/L/sec in VL‐s vs. 0.42 *μ*mol/L/sec in RF‐s and 0.37 *μ*mol/L/sec in RF‐d, *P* < 0.05, Table 2). However, rather than reflecting different muscle fiber type composition related to muscle depth per se these off‐ (and also the on‐) transient deoxygenation profiles might be the consequence of disparate muscle recruitment profiles (Chin et al. 2011). For instance, the overall RF activation (superficial and deep) might be less than that of the VL as suggested by the lower end‐exercise amplitudes of deoxy[Hb + Mb] in the RF‐s and RF‐d versus VL‐s muscle during heavy exercise. This notion is supported by the maximal ramp cycle ergometry data of Chin et al. (2011) who demonstrated that at ~70% of maximal work rate (i.e., heavy exercise herein) the superficial VL achieved close‐to‐peak deoxy[Hb + Mb] whilst the superficial RF was less than 60% of its peak deoxy[Hb + Mb]. However, further investigation is required to determine the range of deoxygenation heterogeneity among deeper compared to superficial muscles.

Exercising muscle temperatures do not differ between the superficial and the deeper region muscles (Kenny et al. 2003) such that regional differences in the Bohr effect would not be expected to impact diffusive O_2_ delivery. However, a greater lactic acidosis in the VL, if present, may assist O_2_ off‐loading and therefore promote elevated deoxy[Hb + Mb].

## Conclusions

Our knowledge of human muscle deoxygenation profiles across the on‐ and off‐transient from heavy exercise is based almost entirely on observations of superficial muscles, most commonly the VL. High power TRS‐NIRS resolves substantially slower deoxy[Hb + Mb] kinetics in the superficial and deep *rectus femoris* muscle compared with the superficial *vastus lateralis* muscle. This original observation indicates a greater reliance on changes in convective O_2_ transport in superficial and deeper RF muscle compared to superficial VL muscle in humans. In addition, a smaller increase and very slow recovery of total[Hb + Mb] in the RF‐s and RF‐d suggests less reliance on changes in diffusive O_2_ transport than seen in VL‐s. The symmetrical pulmonary $\dot{V}O_{2}$ on and off responses belie contrasting O_2_ transport strategies in deeper, more oxidative, muscles compared with the superficial VL that is typically investigated during cycling in humans. This recognition reveals heretofore unappreciated facets of O_2_ transport and metabolic control.

## Conflict of Interest

The authors report no competing interests for this work.
